# Field Resistance to *Phakopsora pachyrhizi* and *Colletotrichum truncatum* of Transgenic Soybean Expressing the *NmDef02* Plant Defensin Gene

**DOI:** 10.3389/fpls.2020.00562

**Published:** 2020-05-26

**Authors:** Natacha Soto, Yuniet Hernández, Celia Delgado, Yamilka Rosabal, Rodobaldo Ortiz, Laura Valencia, Orlando Borrás-Hidalgo, Merardo Pujol, Gil A. Enríquez

**Affiliations:** ^1^Soybean Biotechnology Laboratory, Plant Biotechnology Department, Center for Genetic Engineering and Biotechnology, Havana, Cuba; ^2^National Institute of Agricultural Sciences, San José de las Lajas, Cuba; ^3^Shandong Provincial Key Laboratory of Microbial Engineering, School of Biotechnology, Qilu University of Technology, Jinan, China

**Keywords:** *NmDef02* defensin gene, fungal resistance, *Colletotrichum truncatum*, *Phakopsora pachyrhizi*, soybean, *Bradyrhizobium japonicum*

## Abstract

Fungal diseases lead to significant losses in soybean yields and a decline in seed quality; such is the case of the Asian soybean rust and anthracnose caused by *Phakopsora pachyrhizi* and *Colletotrichum truncatum*, respectively. Currently, the development of transgenic plants carrying antifungal defensins offers an alternative for plant protection against pathogens. This paper shows the production of transgenic soybean plants expressing the *NmDef02* defensin gene using the biolistic delivery system, in an attempt to improve resistance against diseases and reduce the need for chemicals. Transgenic lines were assessed in field conditions under the natural infections of *P. pachyrhizi* and *C. truncatum*. The constitutive expression of the *NmDef02* gene in transgenic soybean plants was shown to enhance resistance against these important plant pathogens. The quantification of the *P. pachyrhizi* biomass in infected soybean leaves revealed significant differences between transgenic lines and the non-transgenic control. In certain transgenic lines there was a strong reduction of fungal biomass, revealing a less severe disease. Integration and expression of the transgenes were confirmed by PCR, Southern blot, and qRT-PCR, where the Def1 line showed a higher relative expression of defensin. It was also found that the expression of the *NmDef02* defensin gene in plants of the Def1 line did not have a negative effect on the nodulation induced by *Bradyrhizobium japonicum.* These results indicate that transgenic soybean plants expressing the *NmDef02* defensin gene have a substantially enhanced resistance to economically important diseases, providing a sound environmental approach for decreasing yield losses and lowering the burden of chemicals in agriculture.

## Introduction

Although soybean [*Glycine max* (L.) Merryll] is one of the most economically important crops worldwide (Hartman et al., 2011; Rosa et al., 2015), its outstanding role in feeding the world through its contribution of both protein meal and vegetable oil, is jeopardized by the attack of fungal diseases at all growth stages, producing a considerable reduction in yields (Hartman et al., 2015). Asian soybean rust caused by *Phakopsora pachyrhizi* (Sydow & Sydow) is the most destructive disease in soybean, causing early defoliation while affecting the weight and quality of the seeds (Hartman et al., 2005). *P. pachyrhizi* can reduce yields by over 80% when environmental conditions are favorable for disease development, and it can affect yields with a disease incidence of just 0.05% (Tremblay et al., 2012). It is found in the soybean-producing countries of South America (Yorinori et al., 2005), the United States (Schneider et al., 2005), and Mexico (Cárcamo Rodríguez et al., 2006). Asian soybean rust has also been reported in other Latin America countries (Yorinori et al., 2005) including Cuba (Pérez-Vicente et al., 2010).

Another important disease that affects soybean is anthracnose produced by *Colletotrichum truncatum*. The soybean plant can be infected at any stage of development (Yang and Hartman, 2015). It is prevalent in tropical and subtropical countries, causing severe effects on grains that lead to seedling loses (Yang and Hartman, 2015; Marmat and Ratnaparkhe, 2017). Furthermore, *C. truncatum* can systemically infect mature plants, and damages are greater under heavy rains with a high plant population (Yang and Hartman, 2015; Pawlowski and Hartman, 2016).

There are now no commercially available soybean cultivars with good agronomic characteristics that are resistant to these diseases. Moreover, modern fungicides cannot effectively control these pathogens, while increasing production costs, and having a strong negative impact on the environment through the use of chemicals (Abdallah et al., 2010; Godoy et al., 2016; Kawashima et al., 2016).

Certain Asian soybean rust resistance genes (*Rpp1-Rpp7*) have been identified in the soybean genome (Lemos et al., 2011; Yamanaka et al., 2013; King et al., 2017; Childs et al., 2018; Hossain and Yamanaka, 2019). However, these genes only confer pathotype-specific resistance, controlled by the interaction of the R genes in soybean with the virulence genes in pathotypes of *P. pachyrhizi* (Vittal et al., 2014; Yamanaka et al., 2015; Langenbach et al., 2016). The effectiveness of specific pathotype resistance genes, whether resistance is complete or incomplete, is usually short-lived, especially when it is evaluated against obligate pathogens such as *P. pachyrhizi* with a high variability and virulence (Yamanaka et al., 2013, 2016). Nevertheless, Kawashima et al. (2016) identified and cloned a gene (*CcRpp1*) from *Cajanus cajan* that confers resistance to different isolates of *P. pachyrhizi* when expressed in soybean.

To counteract fungal infection, plants develop innate immune systems that recognize the presence of pathogens and start effective defense responses (Lay and Anderson, 2005; van Loon et al., 2006). Plants produce pathogenesis-related protein-like defensins (Van Loon, 1997). Defensins are small antimicrobial peptides that play a fundamental role in the innate immunity of plants (Thomma et al., 2002, 2003; van der Weerden et al., 2013; Vriens et al., 2014; Moosa et al., 2018). Their biological activities consist of the inhibition of proteases, blocking of ionic channels, and inhibition of protein synthesis, among others (Graham et al., 2008). Defensins may inhibit the growth of a wide range of microorganisms and phytopathogenic insects; they may also be involved in abiotic stress adaptation (Tavares et al., 2008). This means that not only do defensins produce a defense against plant pathogens, but they also generate adaptations to difficult conditions, a characteristic that makes them even more attractive for modern agriculture. The structure stabilized by disulfide bridges and cationic charge presented by defensins makes them very stable molecules, which is essential for the development of biotechnological products based on them (Tavares et al., 2008).

Growth inhibition by plant defensins of a wide range of pathogenic fungi is not associated with toxicity in mammalian or plant cells (Thomma et al., 2002). Studies of the biological activity, stability, and range of toxicity of an isolated chickpea defensin (Ca-AFP) revealed that there are no risks in using the gene in the production of transgenic crops (Islam, 2008). However, to date, three defensin-related proteins have been described as allergens (Singh et al., 2006; Petersen et al., 2015). It is therefore essential to assess the allergenicity and toxicity of genetically modified crops carrying defensin before they become a product for human and animal use. Consequently, plant defensins can be used to produce transgenic crops with improved resistance to pathogens.

Several genes encoding defensins have been successfully transferred to important plant species such as tobacco (Portieles et al., 2010; Lee et al., 2018), tomato (Abdallah et al., 2010), potato (Gao et al., 2000; Portieles et al., 2010; Kumar and Chakrabarti, 2018), rice (Kanzaki et al., 2002; Jha and Chattoo, 2010), and beans (Espinosa-Huerta et al., 2013), among others, producing resistance to different pathogens. It has been demonstrated that the expression of the *NmDef02* defensin gene in tobacco and potato transgenic plants produced a strong resistance against *Phytophthora infestans* under greenhouse and field conditions (Portieles et al., 2010).

Therefore, our objective in this study was to determine whether transgenic soybean plants expressing the *NmDef02* defensin gene are better equipped to overcome infection by *P. pachyrhizi* and *C. truncatum* under field conditions. The efficiency of the symbiosis of *Bradyrhizobium japonicum* with transgenic soybean plants carrying defensin was also evaluated, since the association with this bacterium is essential for atmospheric nitrogen fixation in soybean plants, eliminating the need for chemical nitrogen fertilization. This strategy is in line with the goals of decreasing yield losses, decreasing the use of chemicals, and contributing to an increase of 100% in yields that are required for sustaining a world population of nearly 10 billion people in 2050, all of which are challenges acknowledged by Next Generation Agriculture.

## Experimental Procedures

### Plant Material

The soybean [*Glycine max* (L.)] was of the variety DT-84 from Vietnam. Embryonic axes of mature seeds were used as explants for their bombardment-mediated transformation, according to Soto et al. (2017).

### Vector Construction

**FIGURE 1:**
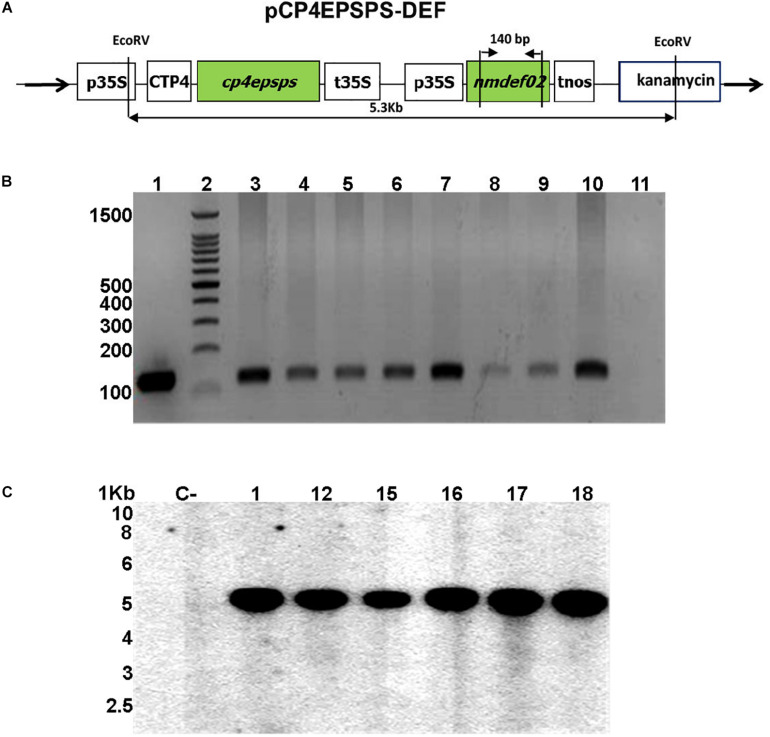
Molecular analyses of transgenic soybean lines. **(A)** Schematic map of the pCP4EPSPS-DEF plasmid used for soybean transformation. The cassette contains the p35S: 35S promoter of the cauliflower mosaic virus, CTP: chloroplast transport peptide, *cp4epsps*: gene that encodes the CP4EPSPS protein, t35S: 35S terminator, *nmdef02*: defensin gene *NmDef02*, tnos: terminator of the nopaline synthase, and two restriction sites of the *Eco*RV enzyme (5.3 Kb). **(B)** PCR reaction products from genomic DNA of soybean plants transformed with the plasmid pCP4EPSPS-DEF. Amplification of a 140 bp sequence corresponding to the *NmDef02* gene. Lane 1: Positive control band of plasmid pCP4EPSPS-DEF; lane 2: 100 bp molecular weight marker (Promega); lanes 3–10: transgenic soybean lines; lane 11: non-transgenic plant (NT). **(C)** Genomic Southern blot analysis of transgenic soybean plants carrying the *Nmdef02* gene. Lane C: non-transgenic control. Lanes 1–18: selected transgenic lines.

The pCP4EPSPS-DEF vector carrying the *cp4epsps* gene and the *NmDef02* defensin gene isolated from *Nicotiana megalosiphon* was the vector system used for the transformation. The cassette with p35S/*NmDef02/tnos* obtained by Portieles et al. (2010) was cloned into the pCP4EPSPS binary vector (Soto et al., 2017) to generate the pCP4EPSPS-DEF vector (Figure 1A). This was done at the soybean biotechnology laboratory at CIGB, Havana.

### Transformation, Selection, and Plant Regeneration

A total of 150 explants were bombarded with the pCP4EPSPS-DEF vector and selected in a MSB5 medium with 20 mg/L of glyphosate. The controls used were explants derived from cultivar DT-84, which were cultured under the same conditions and without selective agent. The regenerated shoots were excised and transferred to the same medium without selecting for rooting, as described by Soto et al. (2017). Plantlets were transferred to pots containing a mixture of organic material and zeolite (50/50) in an acclimatized greenhouse at 26–27°C to produce seeds. All seeds collected from each of the R0 generation transgenic lines were germinated under greenhouse conditions to obtain the T_1_ generation and the following generations.

### Analysis of the Integration of the *NmDef02* Gene Using Polymerase Chain Reaction (PCR)

Total genomic DNA was isolated from young leaves of glyphosate-resistant and control plants using the CTAB protocol (Doyle and Doyle, 1987). PCR was used to screen for transformants (T_1_) carrying the *NmDef02* gene. Each reaction was performed in a total volume of 25 μl, and the PCR mixture consisted of 10 mM buffer Go Taq Green 5x, 10 mM dNTP; 20 pmol/μl of each primer, 1 unit of GoTaq DNA polymerase (Promega, United States), and 400 ng of the genomic DNA. The primers used were forward 5′-GCTGGCTTATGCTTCCTCTTCTTG-3′ and reverse 5′-TCACAGACTTGGACGCAGTTCG-3′. The reaction started with an initial denaturing step at 95°C for 3 min, followed by 30 cycles of the following profile: denaturing at 95°C for 1 min, annealing at 64°C for 1 min, synthesis at 72°C for 1 min followed by an extension at 72°C for 10 min. The PCR products were loaded onto 2% electrophoresis agarose gel and visualized using ethidium bromide.

### Relative Expression of the *NmDef02* Gene Using qRT-PCR

Total RNA for the qPCR analysis was extracted from frozen leaf tissues of six soybean transgenic lines and non-transgenic plants using Tri-Reagent (Sigma-Aldrich, United States) according to the manufacturer’s protocol. The RNA was sequentially treated with DNase I (Promega, United States) at 37°C for 15 min to remove the remaining genomic DNA. The integrity and yield of RNA were evaluated using agarose gel electrophoresis and a NanoDrop Spectrophotometer (Thermo Scientific), respectively. The cDNA was synthesized from 1 μg of total RNA using an oligo-(dT) primer and the Super-Script III reverse transcriptase kit (Invitrogen, United States) according to the manufacturer’s instructions. qPCR reactions were carried out in a final volume of 15 μl containing 0.2 mM of each primer, 10 μl SYBR (QuantiTect SYBR Green PCR kit; Qiagen, Germany), and a dilution of the 25x cDNA. The soybean β*-actin* gene was selected as the housekeeping gene and used for normalizing the data. The primer sequences to amplify the *NmDef02* gene were forward 5′-AAGCTTATGCGTGAGTGCAAGGCTC- 3′ and reverse 5′-CTGCAGTTAGCACTCGAATATAC-3′. The primer sequences from β-actin gene were forward 5′-GTGTCAGCCATACTGTCCCCATTT-3′ and reverse 5′-GTTTCAAGCTCTTGCTCGTAATCA-3′. The amplification conditions included: an initial 95°C denaturation step for 15 min, followed by denaturation for 15 s at 95°C, annealing for 30 s at 60°C, and extension for 30 s at 72°C for 40 cycles. Quantitative PCR was conducted using a Rotor-Gene 3000 PCR machine (Corbett, Sydney, NSW, Australia). The efficiency of the primers was determined by using serial dilutions of a mixture of different cDNAs (from each sample) with concentrations of 5x, 25x, 125x, and 625x. Further analysis of the dissociation temperature of the PCR products was performed to determine their specificity. The dissociation analysis and the Ct values were used by the Rotor-Gene equipment program (version 6.1) to determine the efficiency of the qPCR reactions.

The q-gene method was used to obtain the relative expression of the qPCR values, and they were analyzed with the Q-Gene 96 program (Muller et al., 2002). The results represent the mean of three biological and technical replicates on each transgenic line and the non-transgenic control. The amplified products were sequenced to verify their identity.

### Southern Blot Analysis

Southern blot and hybridization were performed by following the protocol described by Sambrook et al. (1989). Genomic DNA (15 μg) from soybean plants (T4 generation) selected with glyphosate and evaluated in the field was digested with *Eco*RV. The digested DNA was electrophoresed on a 0.8% agarose gel and blotted onto a nylon membrane (Hybond N, Amersham Biosciences). Hybridization was carried out with a-[32P]-dATP-labeled *cp4epsps* gene as the probe, using the DNA random primer labeling kit (Promega, United States). The probe was obtained by PCR with *cp4epsps*-gene-specific primers to generate the 887 bp fragment. It was isolated from a 1% agarose gel and purified using the SV Gel Wizard Clean-Up System (Promega, United States).

### Symbiosis of *Bradyrhizobium japonicum* With Transgenic Soybean Plants Expressing the *NmDef02* Defensin

Because of the essential role of *B. japonicum* in atmospheric nitrogen fixation in soybean, we determined the efficiency of its symbiosis with transgenic soybean plants carrying defensin. The test was performed with 30 transgenic plant seeds and 20 non-transgenic seeds, which were used as the control. The Semia 5080 strain of *B. japonicum* was used for inoculations. Seeds were planted in pots with zeolite and placed in plastic trays with water to maintain humidity within a greenhouse. A week after seed germination, seedlings were inoculated with 1 mL of the diluted bacterial culture at 2 × 10^6^ viable cells/pot. Uninoculated transformed and non-transformed seedlings were used for the control of the assay. The plants were collected during flowering and the following symbiotic efficiency indicators were quantified: number of nodules per plant, fresh weight of nodules per plant (g), fresh leaf weight per plant (g), and dry leaf weight per plant (g). There were three replicates of the experiment.

### *Phakopsora pachyrhizi* Field Trials

Thirteen transgenic lines obtained by self-pollination (T_3_) and non-transgenic plants (variety DT-84) were grown on an experimental area in Havana during the winter (November–March). The field experiment was authorized by the National Center for Biological Safety of Cuba with the license: LH47-L (95) 13. Seeds were inoculated with *B. japonicum* and planted in a field near soybean plants affected by *P. pachyrhizi*. A randomized block design was used, with three blocks/line and 450 seeds/line. Plants were not treated with fungicidal products. The experiment was assessed daily. After the outbreak of rust symptoms, affected leaves were analyzed using an Envirologix QuickStix kit (Envirologix, United States) to confirm the presence of *P. pachyrhizi*.

The incidence of *P. pachyrhizi* was calculated by dividing the number of plants showing symptoms by the total number of plants in the experiment and multiplying the resulting value by 100. When the first symptoms of Asian soybean rust appeared, the severity of the disease (% of the area of the leaf affected by rust) was calculated according to the protocol proposed by Ploper et al. (2006), through which the central folioles of the lower, middle, and upper parts of the plants were sampled. The following scale was used to calculate severity: Grade 1 (0%); Grade 1.5 (0.6–1%); Grade 2 (1–5%); Grade 3 (6–25%); Grade 4 (26–50%); Grade 5 (>50%). The second evaluation took place 10 days after the outbreak, and 20–30 plants were analyzed. The percentage of defoliated plants was calculated at 36 and 60 days after the start of symptoms. Plants were harvested and the following morpho-agronomic parameters of 30 plants for each line were evaluated: height of the plant (cm), height of the 1st pod (cm), number of branches, number of pods, number of seeds, and weight of seeds/plant (g).

#### *Phakopsora pachyrhizi* Biomass

Quantitative PCR was used to measure the fungal biomass of *P. pachyrhizi* in leaves as described by Lamour et al. (2006). Two folioles (from the upper and lower parts of the plants) were collected from each transgenic and non-transgenic plant 10 days after Asian soybean rust symptoms were observed. Thirty plants from each transgenic line and the non-transgenic control were used in this analysis. The collected plant material was frozen at −80°C. The leaves from each line and from the control were pooled separately, macerated in liquid nitrogen, and homogenized to use 1 g of tissue.

Genomic DNA was extracted from frozen leaf tissues of transgenic and non-transgenic plants using a modified CTAB protocol (Doyle and Doyle, 1987). The integrity and yield of DNA were evaluated using agarose gel electrophoresis and a NanoDrop Spectrophotometer (Thermo Scientific), respectively. The qPCR reactions were carried out in a final volume of 20 μl containing 200 ng DNA, 0.8 mM of each primer, and 10 μl SYBR (QuantiTect SYBR Green PCR kit; Qiagen, Germany) by using a Rotor-Gene 3000 PCR machine (Corbett, Sydney, NSW, Australia). The specific primers to amplify an ITS sequence of *P. pachyrhizi* were Ppm1 5′-GCAGAATTCAGTGAATCATCAAG-3′ forward and Ppa4 5′-TCAAAATCCAACAATTTCCC-3′ reverse (Frederick, 2006). The amplification conditions used included: an initial 95°C denaturation step for 15 min, followed by denaturation for 15 s at 95°C, annealing for 30 s at 50°C, and extension for 30 s at 72°C for 40 cycles.

For the quantification of biomass, a standard curve (1/10, 1/100, 1/1000) was made with DNA isolated from pustules of the fungus of highly infested plants. Data were analyzed in Rotor-Gene 3000 software (Corbett). The amplified products were sequenced to verify their identity.

### *Colletotrichum truncatum* Field Trials

Three transgenic lines obtained by self-pollination (T_4_) that were selected for resistance to Asian soybean rust and non-transgenic plants (susceptible variety DT-84) were grown on an experimental area of the National Institute of Agricultural Sciences (INCA), Mayabeque province, during the winter (November–March). Seeds were inoculated with *B. japonicum* and planted in soil with a history of a high incidence of anthracnose caused by *C. truncatum*. A total of 360 seeds from each line and 200 seeds from the non-transgenic plants were used in this study. A randomized block design was used, with three blocks per line. Plants were not treated with fungicidal products, and they were evaluated weekly. After the outbreak of symptoms, infected pod samples were collected and the fungus was isolated and identified (Chen et al., 2006). The incidence of *C. truncatum* in the experiment was calculated by dividing the number of plants with symptoms by the total number of plants in the experiment and multiplying the result by 100. Plants were harvested and the morpho-agronomic parameters of 30 plants of each line were evaluated, specifically the height of the plant (cm), height of the 1st pod (cm), number of branches, number of pods, number of seeds, and weight of seeds/plant (g). Soybean plants transformed with the pCP4EPSPS-DEF (Figure 1A) and pCP4EPSPS (Soto et al., 2017) plasmids were also grown in disease-free soil, using non-transgenic plants (DT-84) as the control. Plants were harvested and the morpho-agronomic parameters of 30 plants were evaluated.

### Statistical Analysis

Data were statistically analyzed by IBM SPSS Statistics 25 using ANOVA at the *P* ≤ 0.05 level. The means of the experimental replicates were plotted, and the standard deviations are shown as error bars.

## Results

### Transformation and Plant Regeneration

Particle acceleration-mediated transformation was carried out using a pCP4EPSPS-DEF vector carrying the glyphosate resistance gene and the *NmDef02* defensin gene under the control of the cauliflower mosaic virus 35S promoter (Figure 1A). The first herbicide-resistant shoots from explants via direct organogenesis were observed after 15 days in the selection medium. Data obtained in the transformation experiment showed that 19 out of 150 bombarded explants developed shoots in the selection medium with glyphosate. All transgenic lines showed similar growth to the non-transformed control and were transferred to greenhouse conditions until the T_2_ seeds were harvested. After having developed their second trifoliate leaf, the plants were sprayed with a concentration of 360 g/L of glyphosate for resistant plant selection. In addition, the expression of the CP4 EPSPS protein was demonstrated in 22 rooted lines (T_0_) using the Roundup Ready immunodetection kit.

### Integration of Transgene in Soybean Plants

In order to analyze the stability of transgene integration in the T_1_ generation, DNA of glyphosate-resistant lines underwent PCR analysis. This analysis detected the presence of the expected 140 bp fragment (Figure 1B), indicating the presence of the *NmDef02* gene in the transgenic soybean plants, while it was not detected in non-transformed plants.

Six transgenic lines selected in glyphosate and showing resistance in the field were screened by Southern blot analysis in generation T_3_. Signals corresponding to the region of the plasmid between the two sites that were recognized by the *Eco*RV enzyme were detected (Figure 1A). The signals showed a stable integration of the segment of the plasmid containing the *cp4epsps* and *NmDef02* genes in the genome of the transformed plants of a size of 5.3 Kb (Figure 1C). DNA isolated from non-transformed plants did not show any hybridization signal (Figure 1C).

### Relative Expression of the *NmDef02* Defensin Gene in Transgenic Plants

**FIGURE 2:**
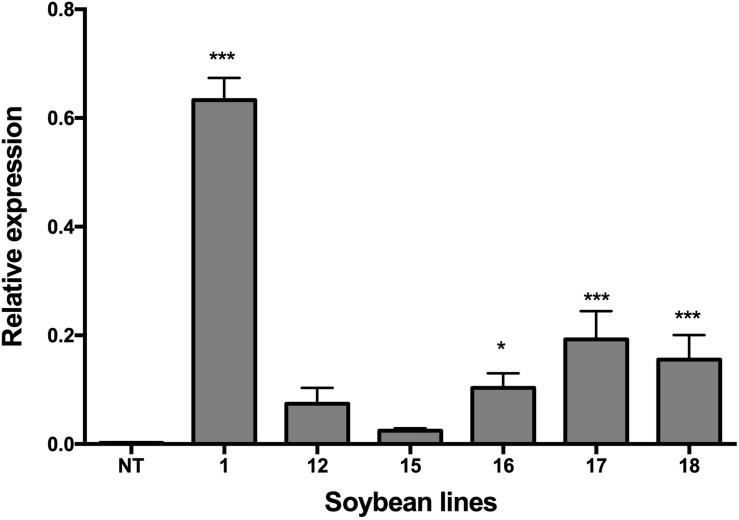
qRT-PCR analyses of transgenic lines showing the transcript levels of the *NmDef02* gene in transgenic plants, compared to the constitutive expression of the endogenous β*-actin* of soybean. 1–18: Transgenic lines (Def1, 12, 15, 16, 17, and 18). NT: non-transgenic control. Bars represent mean (*n* > 9) and standard error of the results obtained. Asterisks indicate significant differences using ANOVA by Tukey’s multiple range test with respect to the control (*p ≤ 0.05, ***p ≤ 0.001).

The relative expression of the *NmDef02* gene in six transgenic soybean lines was evaluated by quantitative RT-PCR (Figure 2). The transgenic lines differed in defensin expression level. Although lines Def1, 17, and 18 showed significant difference (*p* ≤ 0.001) compared to the non-transgenic control, Def1 showed the highest accumulation of defensin NmDef02.

### Efficient Nodulation by *Bradyrhizobium japonicum* in Soybean Plants That Express the *NmDef02* Defensin

**FIGURE 3:**
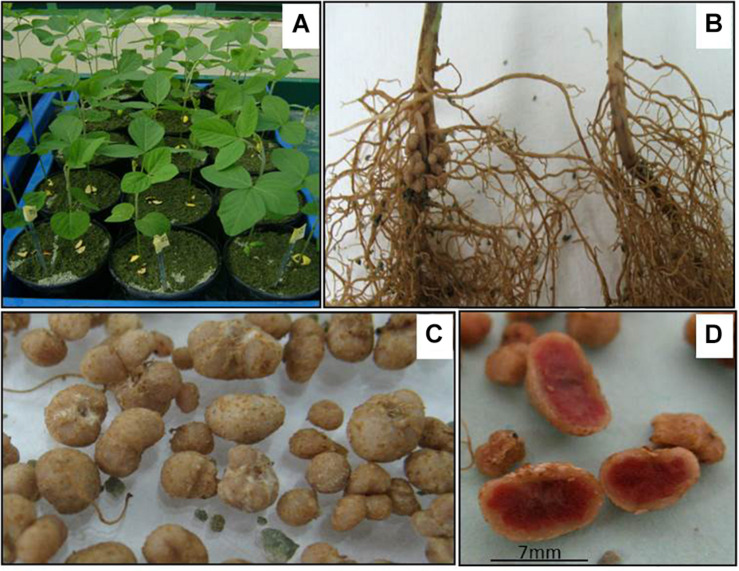
Symbiosis of *Bradyrhizobium japonicum* with soybean plants carrying the *NmDef02* defense gene. **(A)** Transgenic plants in pots with zeolite and inoculated with *B. japonicum*. **(B)** Transgenic plant with nodules at the base of the stem, induced by inoculation (left) and plants without inoculation (right). **(C)** Nodules produced in inoculated transgenic plants. **(D)** Nodular mass with an internal red coloration indicating the production of the leg hemoglobin required for the fixation of atmospheric nitrogen.

This test was carried out to evaluate the efficiency of the symbiosis of this bacterium with transgenic soybean plants carrying defensin. Taking into account that the Def1 transgenic line showed fungal resistance in the experiments performed under natural infection conditions, nodulation in these plants when inoculated with *B. japonicum* was evaluated. In this experiment, the transgenic plants showed a similar phenotypic development to the non-transformed plants used as a control, which was observed in the growth of the stem and color of the leaves, as well as in flowering under greenhouse conditions (Figure 3A). Inoculation produced nodulation in all transgenic plants and the control (Figure 3B). Nodes of different sizes were obtained in all of the inoculated plants (Figure 3C), and the highest number of nodules was observed in the plants harboring defensin, but this did not lead to marked differences in the average fresh weight of the nodules (Table 1). In all cases, the nodules were widely distributed in the root neck region (Figure 3B) and showed an internal red coloring (Figure 3D) due to the presence of the leg-hemoglobin protein. The number of functional nodules with a red coloring in the plants (Table 1) is an indirect indicator of the occurrence of the process of atmospheric nitrogen fixation by the bacteroide. The inoculated plants maintained an intense green color during the experiment and showed higher values of fresh weight and dry weight of the aerial parts than the non-inoculated plants. It is thus shown that the *NmDef02* defensin does not affect *B. japonicum* nodulation.

**TABLE 1:** Nodulation in transgenic and non-transgenic soy plants (cultivate DT-84) inoculated with *Bradyrhizobium japonicum*.

Treatment	Number of nodules	Weight of fresh nodules (g)	Fresh weight of the aerial parts (g)	Dry weight of the aerial parts (g)
**NT**	0 ± 0	0 ± 0	5.5 ± 0.9^b^	1.8 ± 0.6^b^
**NT + *B. japonicum***	8.9 ± 5.8^a^	0.2 ± 0.1	8.2 ± 1.5^a^	2.2 ± 0.5^a^
**Def 1**	0 ± 0	0 ± 0	6.3 ± 1.1^b^	1.9 ± 0.4^b^
**Def 1 + *B. japonicum***	12.4 ± 7^a^	0.2 ± 0.1	7.8 ± 1.7^a^	2.2 ± 0.5^a^

*Def1 (transgenic line) *n* = 30; NT (non-transgenic control DT-84) *n* = 20. The values represent the means ± standard deviation corresponding to three replicates. Different letters show significant differences of *p* < 0.05 according to Tukey’s multiple range test.*

### Field Resistance to *Phakopsora pachyrhizi* in Transgenic Lines

**FIGURE 4:**
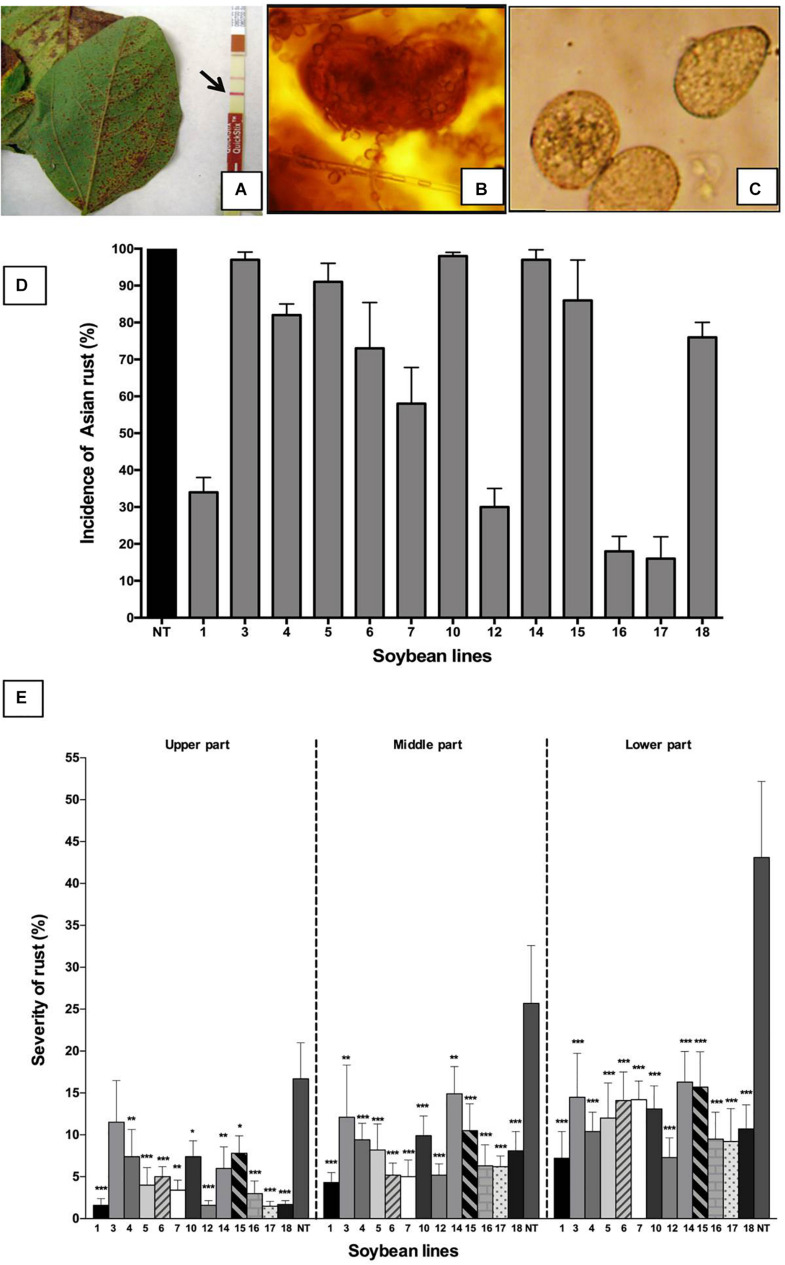
Impact of Asian soybean rust on soybean plants. **(A)** Symptoms of soybean rust in soybean plants. Detection of *P. pachyrhizi* using the Envirologix QuickStix immunodetection strips. Arrow indicates a positive line. **(B)** Urediosoros with uredospore visualized under an optical microscope (20X). **(C)** Uredospore observed under an optical microscope (40X). **(D)** Incidence of soybean rust in soybean plants. The percentages of plants affected by the *P. pachyrhizi* fungus in the transgenic lines (1–18) and in the non-transgenic control (NT) are shown. Bars represent the deviation of the means (*n* = 3). **(E)** Evaluation of soybean rust severity in transgenic soybean plants. The data show the average of two experiments. Severity was determined in different parts of the plant (upper, middle, and lower parts) (*n* = 20). Asterisks indicate significant differences using ANOVA by Tukey’s multiple range test compared to the control (*p ≤ 0.05, **p ≤ 0.01, ***p ≤ 0.001).

After the beginning of Asian soybean rust symptoms in the pod and grain formation stages, the presence of *P. pachyrhizi* was confirmed by the Envirologix QuickStix kit (Figure 4A), and reproductive structures were observed by a stereoscope (Figures 4B,C). Non-transgenic plants (DT-84) used as the control had a 100% incidence of rust (percentage of plants with pustules), demonstrating the high susceptibility of this cultivar to *P. pachyrhizi* (Figure 4D). In parallel, all transgenic lines showed signs of rust; in this case, the pustules were present in the leaves that were closest to the soil, although the percentage of affected plants in transgenic lines was lower than in non-transformed plants (Figure 4D). In this study, some transgenic plants presented a high incidence (Figure 4D) and low severity (Figure 4E) of Asian soybean rust. This was evident in line 18. This is so because, in this study, the incidence only showed the dispersion of fungi in the experimental area. All plants with any symptoms at all were counted, even those with few pustules at the lowest part of the plant, as occurred in lines Def1, 12, 17, and 18. The severity of soybean leaf rust was estimated through the visual observation of plants using the scale proposed by Ploper et al. (2006). The results are shown in Figure 4E. According to this study, severity was higher in the lower parts of the plants, mainly in older trifoliate leaves. All transgenic lines had a significantly lower severity than the control DT-84 (*p* < 0.05), where more than 40% of the leaves involved were from the lower parts of the plant (Figure 4E). Transgenic lines Def1, 12, 16, 17, and 18 showed severe damage by rust; lines Def1 and Def12 had less than 8% of the leaves affected in the lower plant parts, with less than 5% in the middle and upper parts (Figure 4E). Resistance to *P. pachyrhizi* was determined by visual description of signs and symptoms observed on the soybean leaves in response to infection by Asian soybean rust. The presence of reddish-brown (RB) pustules with different levels of sporulation and without sporulation was observed in the leaves of these transgenic lines. Contrarily, in non-transgenic plants, abundant sporulation in uredinias was observed, which shows the high susceptibility of the DT-84 cultivar to *P. pachyrhizi*. The presence of dark brown pustules with limited sporulation was also evident in transgenic and non-transgenic plants. In our study, transgenic lines expressing the *NmDef02* gene showed different levels of resistance to *P. pachyrhizi*, displaying complete and incomplete resistance in lines Def1, 12, 17, and 18. Line Def12 also showed light brown lesions with reduced sporulation in some plants, suggesting a partial resistance. Some plants of the Def3, 4, 6, 10, and 14 lines also showed light brown lesions with reduced sporulation.

**FIGURE 5:**
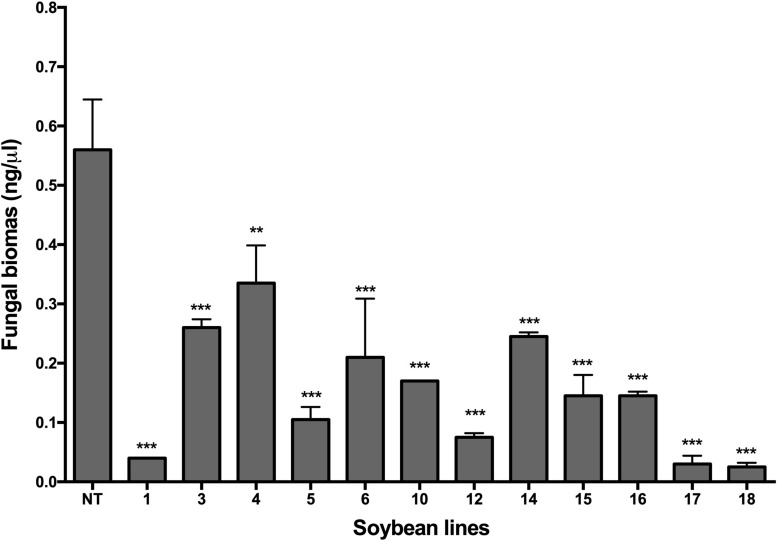
Analysis by qPCR for the quantification of *P. pachyrhizi* DNA in transgenic soybean plants. 1–18: Transgenic lines, and NT: non-transgenic control, DT-84. Bars represent the deviation of the means (*n* = 3). Asterisks indicate significant differences using ANOVA by Tukey’s multiple range test compared to the control (**p ≤ 0.01, ***p ≤ 0.001).

Quantitative PCR analysis confirmed the presence of *P. pachyrhizi*. The quantification of fungal biomass in plants made it possible to verify that all transgenic lines had a significantly lower amount of fungal biomass than the non-transgenic plants (*p* < 0.05), as shown in Figure 5. Transgenic lines Def1, 17, and 18 revealed the lowest amount of fungal biomass compared to the other transgenic lines and the non-transgenic control (Figure 5). The differences in fungal colonization between the transgenic soybean lines were expressed through the quantification of the fungal biomass, even when the visual differences in the signs and symptoms were not evident, as in the cases of lines Def3, 4, 6, and 14.

**FIGURE 6:**
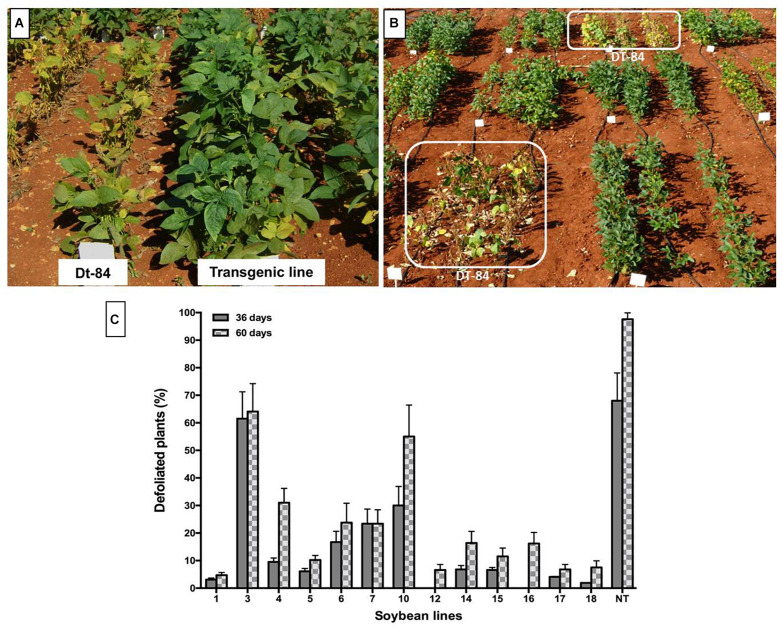
Soybean plants growing in the field and naturally infested with *Phakopsora pachyrhizi* during winter. **(A)** Symptoms of chlorosis by Asian soybean rust in non-transgenic plants (DT-84). **(B)** Symptoms of defoliation in non-transgenic plants due to early maturation. **(C)** Defoliation in soybean plants due to the incidence of Asian soybean rust. Evaluation at 36 and 60 days after the appearance of soybean rust symptoms; 1–18: Transgenic lines, NT: non-transgenic control. Bars represent the deviation of the means (*n* = 3).

Leaves of transgenic plants remained green even when they were affected by *P. pachyrhizi* (Figure 6A), thus contrasting with non-transgenic plants, which showed intense chlorosis in their leaves (Figure 6A) followed by premature defoliation (Figure 6B). The highest percentage of defoliation was observed in non-transgenic plants at 60 days after rust infection. At that time, transgenic lines Def1, 5, 12, 17, and 18 showed defoliation of less than 10% (Figure 6C).

The incidence of rust at this stage caused early defoliation, which had a negative impact on all parameters. The disease affected the number of branches, pods and seeds, as well as the weight of seeds per plant in the non-transgenic control. In contrast, most of the transgenic lines were significantly superior to the control in all parameters related to yield. The results are summarized in Table 2.

**TABLE 2:** Agronomic field test with transgenic soybean lines affected by Asian soybean rust.

Line	Size (cm)	Height of 1st pod (cm)	Number of branches	Number of pods	Number of seeds	Weight of seeds/plant (g)
**Def1**	35,8^bc^	8,4^ab^	2,9^abc^	45,5^ab^	70,7^ab^	9,5^abcd^
**Def3**	30.1^def^	6.8^cd^	1.7^d^	29.1^ef^	50.8^bcd^	7.1^def^
**Def4**	34.3^cd^	7.0^bcd^	2.8^abc^	41.0^abcd^	69.0^abc^	10.3^abc^
**Def5**	22.8^g^	6.3^cd^	2.3^cd^	28.6^ef^	51.4^bcd^	7.7^cde^
**Def6**	25.9^fg^	6.6^cd^	2.3^cd^	29.9^def^	48.5^cd^	6.0^ef^
**Def7**	30.1^def^	6.5^cd^	2.5^bcd^	35.0^bcde^	65.0^abc^	7.2^def^
**Def10**	31.8^cde^	6.2^d^	2.5^bcd^	38.9^abcde^	64.1^abc^	8.6^bcde^
**Def12**	40.9^ab^	7.8^abc^	3.6^a^	39.2^abcde^	73.8^a^	8.1^bcde^
**Def14**	32.2^cde^	6.3^cd^	2.6^bcd^	38.0^abcde^	63.1^abc^	8.3^bcde^
**Def15**	27.4^efg^	6.7^cd^	2.6^bcd^	32.4^cdef^	51.6^bcd^	7.6^cdef^
**Def16**	42.8^a^	9.3^a^	3.5^ab^	38.0^abcde^	60.5^abc^	10.8^ab^
**Def17**	40.8^ab^	7.7^bcd^	3.4^ab^	47.2^a^	81.5^a^	11.7^a^
**Def18**	35.1^dc^	7.7^bcd^	3.1^abc^	43.5^abc^	79.2^a^	11.0^ab^
**NT**	27.6^efg^	6.6^cd^	0.5^e^	22.0^f^	31.2^d^	4.7^f^
**SD**	8.49	1.97	1.38	14.84	27.61	3.84
**SE**	0.41	0.10	0.07	0.72	1.35	0.19

*A simple classification ANOVA was used. Averages with different letters indicate significant differences of *p* < 0.05 according to Tukey’s multiple range test. NT, non-transgenic control; SD, standard deviation; SE, standard error of the means. These data correspond to the average of 30 plants for each line and the non-transgenic control.*

### Resistance to *Colletotrichum truncatum* in Transgenic Lines

**FIGURE 7:**
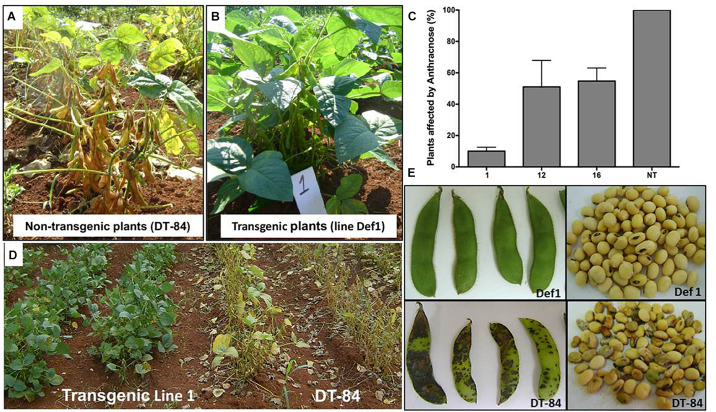
Evaluation of transgenic soybean lines affected by anthracnose (*Colletotrichum truncatum*) in the field experiment. **(A)** Anthracnose symptoms observed in non-transgenic plants cv. DT-84. **(B)** Healthy plants of the Def1 transgenic line. **(C)** Evaluation of the number of plants affected by anthracnose in the field experiment. Transgenic lines (1, 12, and 16). Non-transgenic control (NT). Bars represent the deviation of the means (*n* = 3). **(D)** Early maturity and defoliation in soybean plants. Left: Def1 line, right: non-transgenic control. **(E)** Anthracnose symptoms observed in pods and seeds of non-transgenic plants cv. DT-84. Top: Def1 transgenic line, bottom: non-transgenic control.

A total of 360 transgenic plants carrying the *NmDef02* defensin gene and 200 non-transgenic plants (DT-84) representing the controls were used in the experiment under conditions favoring the incidence of Anthracnose. Symptoms of irregular brown-shaped spots on pods, petioles and stems, similar to those described for anthracnose of soybeans, were observed in non-transgenic plants in the grain formation phase (Figure 7A). Tissues from the affected pods and stems were observed under an optical microscope to examine the structure of the fungus. The presence of *C. truncatum* was confirmed by PCR (data not shown).

In this study, line Def1 showed a high resistance to this pathogen (Figure 7B) because only some plants (10%) (Figure 7C) presented pods with spots at the basal zone of the plant at the end of the cycle. The Def12 and Def16 transgenic lines also showed symptoms of anthracnose, but their incidence was less than in the non-transgenic control (Figure 7C). These transgenic lines showed irregular spots on the pods of some plants, but their leaves remained green until the final stage of plant development, as occurred in line Def1 (Figure 7B). In contrast, 100% of DT-84 plants were affected (Figure 7C) and showed irregular brown spots on pods, as well as leaf chlorosis and high premature defoliation (Figure 7D). On the other hand, the Def12 and Def16 transgenic lines showed a certain reduction in seed quality in some plants. The seeds of line Def1, however, remained healthy (Figure 7E). Seeds and pods of the non-transgenic control were highly affected by the fungus (Figure 7E), showing wrinkling, mold and, in some cases, turning dark brown, which is similar to the symptoms reported for anthracnose associated with Colletotrichum in soybean. After plants were harvested, the results showed a statistically better performance for line Def1 compared to lines Def12 and Def16 and the non-transgenic control in all parameters evaluated (Table 3).

**TABLE 3:** Agronomic field trial with transgenic soybean lines affected by anthracnose.

Line	Size (cm)	Height of 1st pod (cm)	Number of branches	Number of pods	Number of seeds	Weight of seeds/plant (g)
**Def1**	42.28^a^	7.21^a^	3.27^a^	69.57^a^	148.83^a^	29.53^a^
**Def12**	32.60^b^	6.65^ab^	2.67^ab^	39.43^bc^	69.23^bc^	11.60^b^
**Def16**	26.71^c^	6.79^ab^	2.47^b^	40.80^b^	75.83^b^	14.00^b^
**NT**	28.44^c^	6.04^b^	0.53^c^	30.97^c^	55.67^c^	10.25^b^
**SD**	7.31	1.26	1.39	20.35	45.74	10.07
**SE**	0.67	0.12	0.13	1.86	4.18	0.92

*Averages with different letters in the same column indicate significant statistical differences of *p* < 0.05 according to the Tukey’s multiple range test. NT, non-transgenic control. SD, standard deviation. SE, standard error. These data correspond to the average of 30 plants for each line.*

Transgenic soybean lines did not present any detrimental agronomic features compared to the non-transgenic control DT-84 when plants were grown in disease-free soil (Table 4). All soybean plants showed a similar vegetative development. The parameters evaluated show differential behavior, both in transgenic plants carrying the *NmDef02* gene and the *cp4epsp*s gene and in non-transgenic plants, as shown in Table 4. These results demonstrate that the overexpression of defensin did not have a negative impact on parameters related to yield in soybean plants.

**TABLE 4:** Agronomic field trial with transgenic soybean lines under field conditions without fungal diseases.

Lines	Size (cm)	Height of 1st pod (cm)	Number of branches	Number of pods	Number of seeds	Weight of seeds/plant (g)
**1**	40.5^ab^	10.5^b^	2.5^ab^	55.0^a^	126.0^a^	26.6^a^
**17**	40.2^ab^	11.2^b^	3.4^a^	42.7^bc^	99.8^b^	20.1^b^
**18**	40.0^ab^	10.6^b^	2.5^abc^	39.1^bc^	87.1^bc^	20.0^b^
**4**	42.9^a^	14.2^a^	1.9^bcd^	38.2^bc^	79.1^bc^	20.5^b^
**9**	37.5^bc^	10.2^b^	1.2^d^	32.8^c^	63.7^c^	12.9^c^
**12**	32.7^c^	9.7^b^	2.1^bcd^	40.3^bc^	83.1^bc^	18.3^b^
**NT**	43.3^a^	9.6^b^	1.4^cd^	47.9^ab^	94.5^b^	22.1^ab^

*A simple classification ANOVA was performed. Averages with different letters indicate significant differences of *p* < 0.05 according to Tukey’s multiple range test. These data correspond to the average of 30 plants for each transgenic line carrying the *NmDef02* gene (1, 17, 18), the *cp4epsps* gene (4, 9, 12), and the non-transgenic control (NT).*

## Discussion

The production of transgenic plants expressing antimicrobial genes is able to provide broad resistance against different pathogens while reducing the use of chemical pesticides. In the current study, we obtained the first evidence of resistance to the hemibiotrophic fungus *C. truncatum* and the biotrophic fungus *P. pachyrhizi* in soybean plants transformed with the *NmDef02* defensin gene under the 35S constitutive promoter.

Defensin has antifungal activity and produces membrane disruption by pore formation in the cell membrane (Thevissen et al., 2007). A high concentration of defensins produces severe membrane permeabilization, which leads to fungal death (Thevissen et al., 2003; Seo et al., 2014). Previous studies have shown that plant defensins are accumulated in the peripheral cell layers of cotyledons, hypocotyls, endosperm, tubers, fruits, and floral organs, including style, ovary, filaments of stamen, and anthers (Thevissen et al., 2003). These defensin locations are consistent with their role in the first line of defense against potential pathogens (Thevissen et al., 2003; Lay and Anderson, 2005; De Coninck et al., 2013). Plant defensins can also be found in stomatal cells and cell walls of the sub-stomatic cavity; these are involved in plant protection against pathogens that penetrate the stomata (Prema and Pruthvi, 2012).

In this study, we have observed high protection against the pathogenic fungi *P. pachyrhizi* and *C. truncatum* in soybean leaves and pods, which may be favored by the constitutive overexpression of *NmDef02* defensin in the membranes. Previously, Portieles et al. (2010) showed that the constitutive expression of the *NmDef02* defensin gene provided strong resistance to *P. infestans* in transgenic potato plants under greenhouse and field conditions.

The inhibitory activity of defensins on the growth of a wide range of hemibiotrophic and necrotrophic fungi has been observed through *in vitro* studies at micromolar concentrations (Portieles et al., 2010; Lacerda et al., 2016). Nevertheless, studies to determine the antifungal activity of plant defensins against biotrophic fungi are much more difficult, since they are difficult to cultivate *in vitro* according to Kaur et al. (2011). These fungi establish a long-term feeding relationship with the living host cells. Studies recently published by Lacerda et al. (2016), reported that the Drr230a defensin expressed in yeast affected the *in vitro* germination of the spores of the *P. pachyrhizi* fungus. They observed less severity of rust caused by *P. pachyrhizi* in leaves that were artificially inoculated with the fungus and the defensin. Also, the results obtained in our study showed that the transgenic lines expressing the *NmDef02* defensin gene are able to inhibit the development and sporulation of the *P. pachyrhizi* fungus under natural infection conditions. Similar results were reported with the *CcRpp1* gene isolate from *C. cajan* and cloned in soybean, where it conferred specific resistance to *P. pachyrhizi* (Kawashima et al., 2016).

The complex interactions occurring between the pathogen, its host, and the environment are expressed as the incidence or severity of a disease. In this study, the average severity of rust on older leaves (in the lower third of the plants) was statistically higher than on younger leaves (in the upper part of the plants). Similar results were reported by Xavier et al. (2017), who showed that younger soybean plants are more susceptible to Asian soybean rust than older plants but that older trifoliate leaves had the highest disease severity. Although all transgenic lines expressed signs of Asian soybean rust, they were of low severity compared with the non-transgenic control. Studies by Vittal et al. (2014) showed that the presence of reddish-brown pustules (RB) without sporulation indicated complete resistance and that RB lesions with different levels of sporulation meant that there was an incomplete resistance. Both types of lesions were found in the transgenic plants that remained with green leaves, unlike the non-transgenic plants, which showed chlorosis accompanied by the appearance of pustules. On the other hand, the presence of dark brown pustules with limited sporulation in certain transgenic plants shows partial resistance, as described by Pham et al. (2009). Our data support the idea that the constitutive expression of the *NmDef02* gene produced a decrease in the number of pustules in the transgenic lines, and this led to a lower fungal biomass. In contrast, the leaves of the control plants (DT-84) colonized by the pathogen showed a lot of uredias with abundant sporulation and a high amount of *P. pachyrhizi* biomass, demonstrating a compatibility response with the pathogen, as described by some authors (Yamanaka et al., 2010; Vittal et al., 2014). The reduced sporulation of the fungus observed in some plants from the transgenic lines is evidence of resistance to *P. pachyrhizi* in this experiment under natural infection conditions. This effect of the inhibition of fungal germination and growth of spores by plant defensins was also reported in transgenic bean plants that carry the *pdf1.2* defensin gene against *Colletotrichum lindemuthianum* (Espinosa-Huerta et al., 2013).

Plant-pathogen interaction studies have shown that RB lesions can vary in color from light to dark red (Rosa et al., 2015). Because of this, the authors consider that the color of the lesions is not a reliable indicator of resistance or susceptibility to *P. pachyrhizi*. Lesion color is not always a reliable indicator, because it is influenced by environment (Yamanaka et al., 2010). Studies conducted by Miles et al. (2011) also showed that in some cases the severity of the rust is not related to lesion type. However, they found that the number of uredias per leaf area is inversely related to yield (Miles et al., 2011). Similar results were obtained in this study, with different types of lesions on the leaves of transgenic and non-transgenic plants. In addition, the high fungal biomass detected in the plants was related to the large number of uredias present on the leaves, regardless of the type of lesion. It was also consistent with the inverse relationship between severity and yield parameters, where the non-transgenic control plants affected by the fungus had a high percentage of uredias on the leaves and a small number of pods and seeds, as shown in Table 2.

Rust infestation was more severe in certain lines, which also had more fungal biomass on leaves. They were also affected by early defoliation, suggesting that although they were less affected than the DT-84 control, defensin expression was not enough to avoid fungal damage. Studies conducted by Ntui et al. (2010) with the wasabi defensin gene showed that fungal resistance is associated with the level of expression of the protein. In our study, we found a high correlation between the relative expression of *NmDef02*, as determined by qRT-PCR, and the high resistance against *P. pachyrhizi* in transgenic lines Def1, 17, and 18. Interestingly, lines Def12 and 16, which had relatively low expression of defensin, also showed resistance against *P. pachyrhizi* but developed symptoms due to *C. truncatum* infection.

The constitutive expression of *NmDef02* also influenced the proliferation of C. *truncatum*, because there was a decrease in the number of lesions on the transgenic plants, in which line Def1 showed increased resistance to this pathogen. In contrast, DT-84 plants used as controls showed a high susceptibility to *C. truncatum*, with abundant lesions in stems and pods. Similar results were observed in common bean (*Phaseolus vulgaris* L.) carrying the *pdf1.2* defensin gene, where the authors achieved a significant reduction in the formation of lesions in transgenic lines infected with *Colletotrichum* sp. (Espinosa-Huerta et al., 2013).

Some authors state that plants with a short life cycle are able to avoid yield reduction due to Asian soybean rust. This type of mechanism could be a form of horizontal resistance based on an escape mechanism or an unfavorable environment for disease development (Santos et al., 2018). This, however, depends on the susceptibility of the variety and development stage of the plants at the time of the appearance of the pathogen. In the present study, the short cycle cultivar DT-84 used as a non-transgenic control was highly susceptible to *P. pachyrhizi* and *C. truncatum* under the conditions of natural infection, and the plants underwent complete defoliation before concluding their maturation cycle. The early defoliation of these soybean plants reduced productivity by interfering with their physiological processes, thus resulting in less normal pods, fewer seeds per pod and lower grain weight. Disease progression during the pod formation and pod-filling periods is most detrimental to yield (Kawuki et al., 2004). The negative effect of defoliation on crop yields was also observed in Brazilian soybean cultivars affected by Asian soybean rust (da Silva et al., 2015; Childs et al., 2018). This defoliation can affect the natural mechanisms of resistance, making them less active and increasing the susceptibility of soybean to end-of-cycle diseases.

To conclude, in experiments where no chemical fungicides were applied, transgenic plants showed increased resistance to *P. pachyrhizi* and *C. truncatum* until the end-of-cycle stage, where other pathogens normally appear. Molecular analyses showed the presence of the transgene in the progeny of these lines, where transgenic line Def1 accumulated the highest transcript levels and displayed the highest degree of resistance to both diseases. This could explain why severity and incidence of and defoliation by Asian soybean rust in plants of this transgenic line were lower. Similarly, the reduced fungal biomass present in the transgenic plants coincides with a reduced sporulation of the pathogen, which demonstrates the antifungal effect exerted by defensin *NmDef02* on *P. pachyrhizi.*

The antifungal effect of the *NmDef02* defensin had been previously demonstrated by Portieles et al. (2010). However, it is not obvious that there is resistance to a biotrophic fungus such as *P. pachyrhizi*, which is difficult to control, or against *C. truncatum*, because resistance to fungal pathogens is not only obtained by introducing this defensin into a culture. An example of this is the susceptibility to these fungi observed in some transgenic lines evaluated in the field.

Transgenic soybean plants had a similar development to non-transgenic plants when inoculated with *B. japonicum*. The use of rhizospheric microorganisms in the preparation of inoculants for soybeans was very important in maintaining high productivity with a lower environmental impact, as demonstrated by some authors (Menéndez et al., 2014; Nápoles García et al., 2014). This study also showed that the expression of the *NmDef02* defensin gene in soybean plants had no negative effect on the nodulation induced by *B. japonicum*, a bacterium that plays an essential role in the technology of this crop in Cuba. Kaur et al. (2017) studied the symbiosis of mycorrhizae with transgenic wheat plants carrying MtDef4.2 defensin. This study also showed that the expression of that defensin in apoplast can provide resistance to leaf rust, without having a negative effect on the symbiosis with that beneficial fungus.

To the best of our knowledge, this is the first report on the transformation of soybean with a defensin gene for resistance to fungal pathogens. We demonstrated that the overexpression of the *NmDef02* gene resulted in a delay in progression of the fungi. However, more evaluations of these transgenic lines against these pathogens in different weather conditions are necessary, taking into account other parameters that were not taken into account in this study, to confirm resistance. A complete resistance to *P. pachyrhizi* was not found in the transgenic lines, and the application of fungicide is needed to completely control the pathogen. Evidently, the use of transgenic plants expressing this defensin would reduce the number of chemical fungicide applications in the field for an integral pest management in soybean with a minimal environmental impact. These results provide a sound environmental approach to decrease yield losses and to lower the burden of chemicals, both goals targeted by Next Generation Agriculture.

## Data Availability Statement

All datasets generated for this study are included in the article/supplementary material.

## Author Contributions

NS and GE conceived and designed the research work. YR performed the construction of pCP4EPSPS-DEF plasmid. NS, YH, and CD performed the soybean transformation experiments. NS, YH, CD, LV, RO, and GE performed the field experiments. NS, YH, OB-H, and GE conducted molecular analysis and analyzed the data. NS wrote the manuscript. GE, MP, and OB-H reviewed the manuscript.

## Conflict of Interest

The authors declare that the research was conducted in the absence of any commercial or financial relationships that could be construed as a potential conflict of interest.

## References

[B1] Abdallah N. A. Shah D. Abbas D. Madkour M. (2010). Stable integration and expression of a plant defensin in tomato confers resistance to fusarium wilt. *GM Crops* 1 344–350. 10.4161/gmcr.1.5.15091 21844692

[B2] Cárcamo Rodríguez A. Ríos J. A. Hernández J. (2006). First report of Asian soybean rust caused by *Phakopsora pachyrhizi* from Mexico. *Plant Dis.* 90 1260–1260. 10.1094/PD-90-1260B 30781112

[B3] Chen L. Chu C. Liu C. Chen R. Tsay J. (2006). PCR-based detection and differentiation of anthracnose pathogens, colletotrichum gloeosporioides and *C. truncatum*, from vegetable soybean in Taiwan. *J. Phytopathol.* 154, 654–662.

[B4] Childs S. P. Buck J. W. Li Z. (2018). Breeding soybeans with resistance to soybean rust (*Phakopsora pachyrhizi*). *Plant Breed.* 137 250–261.

[B5] da Silva A. F. Sediyama T. dos Santos Silva F. C. Bezerra A. R. G. Rosa D. P. Cruz C. D. (2015). Effect of defoliation from the bottom to the top on the yield of Brazilian soybean cultivars. *Afr. J. Agric. Res.* 10 3296–3304.

[B6] De Coninck B. Cammue B. P. A. Thevissen K. (2013). Modes of antifungal action and in planta functions of plant defensins and defensin-like peptides. *Fungal Biol. Rev.* 26 109–120.

[B7] Doyle J. Doyle J. (1987). CTAB DNA extraction in plants. *Phytochem. Bull.* 19 11–15.

[B8] Espinosa-Huerta E. Quintero-Jiménez A. Sánchez-García B. M. Acosta-Gallegos J. A. Mora-Avilés M. A. (2013). Resistencia a *Colletotrichum lindemuthianum* en frijol común transgénico, expresando el gen defensina de *Arabidopsis thaliana*. *Rev. Mex. Cienc. Agríc.* 4 1027–1042.

[B9] Frederick R. D. (2006). *PCR Methods for the Identification and Detection of the Soybean Rust Pathogen Phakopsora Pachyrhizi.* Washington, DC: US Department of Agriculture.

[B10] Gao A.-G. Hakimi S. M. Mittanck C. A. Wu Y. Woerner B. M. Stark D. M. (2000). Fungal pathogen protection in potato by expression of a plant defensin peptide. *Nat. Biotechnol.* 18 1307–1310. 10.1038/82436 11101813

[B11] Godoy C. V. Seixas C. D. S. Soares R. M. Marcelino-Guimarães F. C. Meyer M. C. Costamilan L. M. (2016). Asian soybean rust in Brazil: past, present, and future. *Pesqui. Agropecu. Brasileira* 51 407–421. 10.1094/PDIS-07-14-0772-SR

[B12] Graham M. Silverstein K. VandenBosch K. (2008). Defensin-like genes: genomic perspectives on a diverse superfamily in plants. *Plant Genome* 48(Suppl. 1), S3–S11.

[B13] Hartman G. L. Miles M. R. Frederick R. D. (2005). Breeding for resistance to soybean rust. *Plant Dis.* 89 664–666. 10.1094/PD-89-0664 30795395

[B14] Hartman G. L. West E. D. Herman T. K. (2011). Crops that feed the World 2. Soybean—worldwide production, use, and constraints caused by pathogens and pests. *Food Security* 3 5–17.

[B15] Hartman G. L. Rupe J. C. Sikora E. J. Domier L. L. Davis J. A. Steffey K. L. eds. (2015). **Compendium of Soybean Diseases and Pests**. St. Paul: Am Phytopath Society, 4–16.

[B16] Hossain M. M. Yamanaka N. (2019). Pathogenic variation of Asian soybean rust pathogen in Bangladesh. *J. Gen. Plant Pathol.* 85 90–100.

[B17] Islam A. (2008). Preliminary risk assessment of a novel antifungal defensin peptide from chickpea (*Cicer arietinum* L.). *Appl. Biosaf.* 13 222–230.

[B18] Jha S. Chattoo B. B. (2010). Expression of a plant defensin in rice confers resistance to fungal phytopathogens. *Trans. Res.* 19 373–384. 10.1007/s11248-009-9315-7 19690975

[B19] Kanzaki H. Nirasawa S. Saitoh H. Ito M. Nishihara M. Terauchi R. (2002). Overexpression of the wasabi defensin gene confers enhanced resistance to blast fungus (*Magnaporthe grisea*) in transgenic rice. *Theor. Appl. Genet.* 105 809–814. 10.1007/s00122-001-0817-9 12582903

[B20] Kaur J. Fellers J. Adholeya A. Velivelli S. L. El-Mounadi K. Nersesian N. (2017). Expression of apoplast-targeted plant defensin MtDef4. 2 confers resistance to leaf rust pathogen *Puccinia triticina* but does not affect mycorrhizal symbiosis in transgenic wheat. *Trans. Res.* 26 37–49. 10.1007/s11248-016-9978-9 27582300 PMC5243879

[B21] Kaur J. Sagaram U. S. Shah D. (2011). Can plant defensins be used to engineer durable commercially useful fungal resistance in crop plants? *Fungal Biol. Rev.* 25 128–135.

[B22] Kawashima C. G. Guimarães G. A. Nogueira S. R. MacLean D. Cook D. R. Steuernagel B. (2016). A pigeonpea gene confers resistance to Asian soybean rust in soybean. *Nat. Biotechnol.* 34:661. 10.1038/nbt.3554 27111723

[B23] Kawuki R. Tukamuhabwa P. Adipala E. (2004). Soybean rust severity, rate of rust development, and tolerance as influenced by maturity period and season. *Crop Protect.* 23 447–455.

[B24] King Z. R. Childs S. P. Harris D. K. Pedley K. F. Buck J. W. Boerma H. R. (2017). A new soybean rust resistance allele from PI 423972 at the Rpp4 locus. *Mol. Breed.* 37:62.

[B25] Kumar M. Chakrabarti S. K. (2018). Expression of β-defensin gene in potato confers enhanced resistance to *Ralstonia Solanacearum* L. *Defence Life Sci. J.* 3 15–23.

[B26] Lacerda A. F. Del Sarto R. P. Silva M. S. de Vasconcelos E. A. R. Coelho R. R. dos Santos V. O. (2016). The recombinant pea defensin Drr230a is active against impacting soybean and cotton pathogenic fungi from the genera Fusarium. Colletotrichum and Phakopsora. *3 Biotech* 6 1–10. 10.1007/s13205-015-0320-7 PMC4752952 28330129

[B27] Lamour K. H. Finley L. Snover-Clift K. L. Stack J. P. Pierzynski J. Hammerschmidt R. (2006). Early detection of Asian soybean rust using PCR. *Plant Health Prog.* 7:20.

[B28] Langenbach C. Schultheiss H. Rosendahl M. Tresch N. Conrath U. Goellner K. (2016). Interspecies gene transfer provides soybean resistance to a fungal pathogen. *Plant Biotechnol. J.* 14 699–708. 10.1111/pbi.12418 26096357 PMC4745023

[B29] Lay F. T. Anderson M. A. (2005). Defensins-components of the innate immune system in plants. *Curr. Protein Peptide Sci.* 6 85–101. 15638771 10.2174/1389203053027575

[B30] Lee H.-H. Kim J.-S. Hoang Q. T. Kim J.-I. Kim Y. S. (2018). Root-specific expression of defensin in transgenic tobacco results in enhanced resistance against *Phytophthora parasitica* var. nicotianae. *Eur. J. Plant Pathol.* 151 811–823.

[B31] Lemos N. G. Braccini A. D. L. Abdelnoor R. V. de Oliveira M. C. N. Suenaga K. Yamanaka N. (2011). Characterization of genes *Rpp*2. *Rpp4*, and *Rpp5* for resistance to soybean rust. *Euphytica* 182:53 10.1007/s00122-017-2983-4

[B32] Marmat N. Ratnaparkhe M. (2017). Molecular and phylogenetic studies of *Colletotrichum truncatum* associated with soybean anthracnose in India. *Plant Pathol. Q.* 7 146–152.

[B33] Menéndez C. Trujillo L. E. Ramírez R. González-Peña D. Espinosa D. Enriquez G. A. (2014). Producción de un inoculante líquido de *Bradyrhizobium japonicum* con alto impacto en la siembra mecanizada de la soya en Cuba. *Biotecnol. Apl.* 31 116–120.

[B34] Miles M. Bonde M. Nester S. Berner D. Frederick R. Hartman G. (2011). Characterizing resistance to *Phakopsora pachyrhizi* in soybean. *Plant Dis.* 95 577–581. 30731946 10.1094/PDIS-06-10-0450

[B35] Moosa A. Farzand A. Sahi S. T. Khan S. A. (2018). Transgenic expression of antifungal pathogenesis-related proteins against phytopathogenic fungi – 15 years of success. *Israel J. Plant Sci.* 65:38 10.1080/07929978.2017.1288407

[B36] Muller P. Y. Janovjak H. Miserez A. R. Dobbie Z. (2002). Short technical report processing of gene expression data generated by quantitative real-time RT-PCR. *Biotechniques* 32 1372–1379. 12074169

[B37] Nápoles García M. C. González-Anta G. Ferreira A. Rossi A. Hernández Forte I. Costales Menéndez D. (2014). Efecto de diferentes inoculantes sobre la nodulación de la soya cultivada en condiciones de estrés. *Cult. Trop.* 35 45–51.

[B38] Ntui V. O. Thirukkumaran G. Azadi P. Khan R. S. Nakamura I. Mii M. (2010). Stable integration and expression of wasabi defensin gene in “Egusi” melon (*Colocynthis citrullus* L.) confers resistance to Fusarium wilt and Alternaria leaf spot. *Plant Cell Rep.* 29 943–954. 10.1007/s00299-010-0880-2 20552202

[B39] Pawlowski M. L. Hartman G. L. (2016). “Infection Mechanisms and Colonization Patterns of Fungi Associated with Soybean,” in *Fungal Pathogenicity*, ed. Sultan S. (London: IntechOpen), 25.

[B40] Pérez-Vicente L. Martínez-de la Parte E. Pérez-Miranda M. Martín-Triana E. Borrás-Hidalgo O. Hernández-Estévez I. (2010). First report of Asian rust of soybean caused by *Phakopsora pachyrhizi* in Cuba. *Plant Pathol.* 59 803–803.

[B41] Petersen A. Kull S. Rennert S. Becker W.-M. Krause S. Ernst M. (2015). Peanut defensins: novel allergens isolated from lipophilic peanut extract. *J. Allergy Clin. Immunol.* 136 1295.–1301. 10.1016/j.jaci.2015.04.010 26037551

[B42] Pham T. Miles M. Frederick R. Hill C. Hartman G. (2009). Differential responses of resistant soybean entries to isolates of *Phakopsora pachyrhizi*. *Plant Dis.* 93 224–228. 10.1094/PDIS-93-3-0224 30764187

[B43] Ploper L. D. Escobar D. Ivancovich A. Diaz C. G. Sillon M. Formento N. (2006). Propuesta de protocolo para muestreo y evaluación de la roya asiática de la soja en Argentina. *Ava. Agroind. Estación Exp. Agro Ind. Obispo Colombres* 27 35–37.

[B44] Portieles R. Ayra C. Gonzalez E. Gallo A. Rodriguez R. Chacón O. (2010). NmDef02, a novel antimicrobial gene isolated from *Nicotiana megalosiphon* confers high-level pathogen resistance under greenhouse and field conditions. *Plant Biotechnol. J.* 8 678–690. 10.1111/j.1467-7652.2010.00501.x 20626828

[B45] Prema G. U. Pruthvi T. P. M. (2012). Antifungal plant defensins. *Curr. Biot.* 6 254–270.

[B46] Rosa C. Spehar C. Liu J. (2015). Asian soybean rust resistance: an overview. *J. Plant Pathol. Microb.* 6:2. 20088771

[B47] Sambrook J. Fritsch E. Maniatis T. (1989). *Molecular Cloning: A Laboratory Manual.* Cold Spring Harbor, NY: Cold Spring Harbor.

[B48] Santos J. V. M. D. Yamanaka N. Marcelino-Guimarães F. C. de Toledo J. F. F. Arias C. A. A. Abdelnoor R. V. (2018). Molecular mapping of quantitative trait loci for agronomical traits in soybean under Asian soybean rust infection. *Crop Breed. App. Biotechnol.* 18 390–398.

[B49] Schneider R. Hollier C. Whitam H. Palm M. McKemy J. Hernandez J. (2005). First report of soybean rust caused by *Phakopsora pachyrhizi* in the continental United States. *Plant Dis.* 89 774–774. 10.1094/PD-89-0774A 30791253

[B50] Seo H.-H. Park S. Park S. Oh B.-J. Back K. Han O. (2014). Overexpression of a defensin enhances resistance to a fruit-specific anthracnose fungus in pepper. *PLoS One* 9:e97936 10.1371/journal.pone.0097936 PMC4029827 24848280

[B51] Singh A. Mehta A. Sridhara S. Gaur S. Singh B. Sarma P. (2006). Allergenicity assessment of transgenic mustard (*Brassica juncea*) expressing bacterial codA gene. *Allergy* 61 491–497. 10.1111/j.1398-9995.2006.01049.x 16512812

[B52] Soto N. Delgado C. Hernández Y. Rosabal Y. Ferreira A. Pujol M. (2017). Efficient particle bombardment-mediated transformation of Cuban soybean (INCASoy-36) using glyphosate as a selective agent. *Plant Cell Tissue Organ Cult.* 128 187–196.

[B53] Tavares L. S. Santos M. D. O. Viccini L. F. Moreira J. S. Miller R. N. Franco O. L. (2008). Biotechnological potential of antimicrobial peptides from flowers. *Peptides* 29 1842–1851. 10.1016/j.peptides.2008.06.003 18602431

[B54] Thevissen K. Ferket K. K. François I. E. Cammue B. P. (2003). Interactions of antifungal plant defensins with fungal membrane components. *Peptides* 24 1705–1712. 10.1016/j.peptides.2003.09.014 15019201

[B55] Thevissen K. Kristensen H. H. Thomma B. P. Cammue B. P. Francois I. E. (2007). Therapeutic potential of antifungal plant and insect defensins. *Drug Discov. Today* 12 966–971. 10.1016/j.drudis.2007.07.016 17993416

[B56] Thomma B. P. Cammue B. P. Thevissen K. (2002). Plant defensins. *Planta* 216 193–202. 12447532 10.1007/s00425-002-0902-6

[B57] Thomma B. P. Cammue B. P. Thevissen K. (2003). Mode of action of plant defensins suggests therapeutic potential. *Curr. Drug Targets Infect. Disord.* 3 1–8. 10.2174/1568005033342000 12570728

[B58] Tremblay A. Hosseini P. Li S. Alkharouf N. W. Matthews B. F. (2012). Identification of genes expressed by *Phakopsora pachyrhizi*, the pathogen causing soybean rust, at a late stage of infection of susceptible soybean leaves. *Plant Pathol.* 61 773–786.

[B59] van der Weerden N. L. Bleackley M. R. Anderson M. A. (2013). Properties and mechanisms of action of naturally occurring antifungal peptides. *Cell. Mole. Life Sci.* 70 3545–3570. 10.1007/s00018-013-1260-1 PMC11114075 23381653

[B60] Van Loon L. (1997). Induced resistance in plants and the role of pathogenesis-related proteins. *Eur. J. Plant Pathol.* 103 753–765.

[B61] van Loon L. C. Rep M. Pieterse C. M. (2006). Significance of inducible defense-related proteins in infected plants. *Annu. Rev. Phytopathol.* 44 135–162. 10.1146/annurev.phyto.44.070505.143425 16602946

[B62] Vittal R. Paul C. Hill C. Hartman G. (2014). Characterization and quantification of fungal colonization of *Phakopsora pachyrhizi* in soybean genotypes. *Phytopathology* 104 86–94. 10.1094/PHYTO-12-12-0334-R 24073640

[B63] Vriens K. Cammue B. Thevissen K. (2014). Antifungal plant defensins: mechanisms of action and production. *Molecules* 19 12280–12303. 10.3390/molecules190812280 25153857 PMC6271847

[B64] Xavier S. A. Martins D. C. Fantin L. H. Canteri M. G. (2017). Older leaf tissues in younger plants are more susceptible to soybean rust. *Acta Scientiarum Agron.* 39 17–24.

[B65] Yamanaka N. Lemos N. G. Uno M. Akamatsu H. Yamaoka Y. Abdelnoor R. V. (2013). Resistance to Asian soybean rust in soybean lines with the pyramided three Rpp genes. *Crop Breedi. Appl. Biotechnol.* 13 75–82.

[B66] Yamanaka N. Morishita M. Mori T. Lemos N. G. Hossain M. M. Akamatsu H. (2015). Multiple Rpp-gene pyramiding confers resistance to Asian soybean rust isolates that are virulent on each of the pyramided genes. *Trop. Plant Pathol.* 40 283–290.

[B67] Yamanaka N. Morishita M. Mori T. Muraki Y. Hasegawa M. Hossain M. (2016). The locus for resistance to Asian soybean rust in PI 587855. *Plant Breed.* 135 621–626.

[B68] Yamanaka N. Yamaoka Y. Kato M. Lemos N. G. Passianotto A. L. D. L. dos Santos J. V. (2010). Development of classification criteria for resistance to soybean rust and differences in virulence among Japanese and Brazilian rust populations. *Trop. Plant Pathol.* 35 153–162.

[B69] Yang H.-C. Hartman G. L. (2015). Methods and evaluation of soybean genotypes for resistance to *Colletotrichum truncatum*. *Plant Dis.* 99 143–148. 10.1094/PDIS-03-14-0228-RE 30699740

[B70] Yorinori J. Paiva W. Frederick R. Costamilan L. Bertagnolli P. Hartman G. (2005). Epidemics of soybean rust (*Phakopsora pachyrhizi*) in Brazil and Paraguay from 2001 to 2003. *Plant Dis.* 89 675–677. 10.1094/PD-89-0675 30795398

